# Involvement in treatment decision-making and self-reported efficacy among patients with advanced colorectal cancer: a nationwide multi-center cross-sectional study

**DOI:** 10.3389/fonc.2023.1168078

**Published:** 2023-07-26

**Authors:** Xiao-Fen Gu, Hui-Fang Xu, Yin Liu, Li Li, Yan-Qin Yu, Xi Zhang, Xiao-Hui Wang, Wen-Jun Wang, Ling-Bin Du, Shuang-Xia Duan, He-Lu Cao, Yu-Qian Zhao, Yun-Yong Liu, Juan-Xiu Huang, Ji Cao, Yan-Ping Fan, Chang-Yan Feng, Xue-Mei Lian, Jing-Chang Du, Remila Rezhake, Li Ma, You-Lin Qiao

**Affiliations:** ^1^ Department of Student Affairs, Affiliated Cancer Hospital of Xinjiang Medical University, Urumqi, China; ^2^ Department of Cancer Epidemiology, Affiliated Cancer Hospital of Zhengzhou University & Henan Cancer Hospital, Henan Engineering Research Center of Cancer Prevention and Control, Henan International Joint Laboratory of Cancer Prevention, Zhengzhou, China; ^3^ Department of Clinical Research, The First Affiliated Hospital of Jinan University, Guangzhou, Guangdong, China; ^4^ The Clinical Epidemiology of Research Center, Department of Public Health and Preventive Medicine, Baotou Medical College, Baotou, China; ^5^ Key Laboratory of Carcinogenesis and Translational Research (Ministry of Education/Beijing), Beijing Office for Cancer Prevention and Control, Peking University Cancer Hospital & Institute, Beijing, China; ^6^ Department of Public Health, Gansu Provincial Cancer Hospital, Lanzhou, China; ^7^ School of Nursing, Jining Medical University, Jining, China; ^8^ Department of Cancer Prevention, The Cancer Hospital of the University of Chinese Academy of Sciences, Zhejiang Cancer Hospital, Hangzhou, China; ^9^ Department of Preventive Health, Xinxiang Central Hospital, Xinxiang, China; ^10^ Center for Cancer Prevention Research, Sichuan Cancer Hospital & Institute, Sichuan Cancer Center, School of Medicine, University of Electronic Science and Technology of China, Chengdu, China; ^11^ Liaoning Office for Cancer Control and Research, Cancer Hospital of China Medical University, Liaoning Cancer Hospital and Institute, Shenyang, China; ^12^ Department of Gastrodiges, Wuzhou Red Cross Hospital, Wuzhou, China; ^13^ Department of Cancer Prevention and Control Office, the First Affiliated Hospital of Guangxi Medical University, Nanning, China; ^14^ State Key Laboratory of Oncology in South China, Collaborative Innovation Center for Cancer Medicine, Sun Yat-Sen University Cancer Center, Guangzhou, China; ^15^ Chongqing Key Laboratory of Translational Research for Cancer Metastasis and Individualized Treatment, Chongqing University Cancer Hospital, Chongqing, China; ^16^ School of Public Health and Management, Chongqing Medical University, Chongqing, China; ^17^ School of Public Health, Chengdu Medical College, Chengdu, China; ^18^ Public Health School, Dalian Medical University, Dalian, China; ^19^ School of Population Medicine and Public Health, Chinese Academy of Medical Sciences and Peking Union Medical College, Beijing, China

**Keywords:** colorectal cancer, treatment, decision making, self-reported efficacy, China

## Abstract

**Introduction:**

This cross-sectional study evaluated the involvement of patients with advanced colorectal cancer (CRC) in treatment decision-making, assessed the treatment efficacy according to their self-reports, and investigated the influencing factors.

**Methods:**

Patients with advanced CRC were recruited from 19 hospitals from March 2020 to March 2021 by a multi-stage multi-level sampling method. A self-designed questionnaire was used to collect demographic and clinical characteristics, involvement of CRC patients in treatment decision-making, treatment methods, and self-reported efficacy. Univariate and unordered multinomial logistic regression analyses were used to evaluate the factors affecting the involvement in treatment decision-making and self-reported efficacy.

**Results:**

We enrolled 4533 patients with advanced CRC. The average age at diagnosis was 58.7 ± 11.8 years. For the treatment method, 32.4% of patients received surgery combined with chemotherapy, 13.1% of patients underwent surgery combined with chemotherapy and targeted therapy, and 9.7% of patients were treated with surgery alone. For treatment decision-making, 7.0% of patients were solely responsible for decision-making, 47.0% of patients shared treatment decision-making with family members, 19.0% of patients had family members solely responsible for treatment decision-making, and 27.0% of patients had their physicians solely responsible for treatment decision-making. Gender, age, education level, family income, marital status, treatment cost, hospital type, and treatment method were significantly associated with the involvement of patients in treatment decision-making. A total of 3824 patients submitted self-reported efficacy evaluations during treatment. The percentage of patients with good self-reported efficacy was 76.5% (for patients treated for the first time), 61.7% (for patients treated for the second time), and 43.2% (for patients treated after recurrence and metastasis), respectively. Occupation, education level, average annual family income, place of residence, time since cancer diagnosis, hospital type, clinical stage, targeted therapy, and involvement in treatment decision-making were the main influencing factors of self-reported efficacy of treatment.

**Discussion:**

Conclusively, CRC patients are not highly dominant in treatment decision-making and more likely to make treatment decisions with their family and doctors. Timely and effective communication between doctors and patients can bolster patient involvement in treatment decision-making.

## 1 Introduction

Colorectal cancer (CRC) is one of the most common malignancies worldwide, with morbidity ranking third and mortality ranking second. More than half of new cases and CRC-related deaths are from China, Europe, and North America (1). It is estimated that approximately 1.9 million newly diagnosed CRC cases and 935,000 CRC-related deaths in 2020, accounting for approximately one-tenth of new cancer cases and deaths (2). In recent years, the morbidity and mortality of CRC have decreased in some countries in Europe and the United States. However, China is still suffering a remarkable CRC burden, which accounts for 28.11% of global deaths. In China, both men and women have higher crude mortality rates for CRC than the global average. Moreover, the prevalence of CRC is on the rise in China (3, 4).

In 2015, there were about 387,600 new CRC cases and 187,100 CRC-related deaths, accounting for 9.87% and 8.01% of all malignant tumors, respectively, in China (5, 6). In recent years, with the implementation of screening, early diagnosis, and treatment in China, the age-standardized mortality of CRC has decreased from 10.01/100,000 in 2005 to 9.68/100,000 in 2020, and the 5-year survival rate has increased from 47.2% in 2003-2005 to 56.9% in 2012-2015. However, it is worth noting that more than half of the patients have advanced CRC at initial diagnosis (7), with a 5-year survival rate of approximately 20% (8). It has been shown that there are significant differences in the prognosis of CRC between different treatment methods, countries, or regions (9, 10). Moreover, satisfaction and compliance with treatment may be improved by the involvement of patients in treatment decision-making, thereby indirectly prompting outcomes (11). Therefore, the involvement of patients in treatment decisions making and further analysis of factors associated with the prognosis of advanced CRC is important for making individual diagnoses and treatment plans and for improving compliance and treatment efficacy, further reducing disease burden.

Previously, most of the studies on CRC were designed as single-centered (12) or focused on specific subjects in a certain clinical stage (13), the results of which could not be generalized. Generally, patients have high expectations of participating in treatment decision-making, but the actual involvement is low (14). Moreover, there are rare studies on the involvement of CRC patients in diagnosis and treatment decision-making. Additionally, several prognostic factors of CRC have been reported, such as distant metastasis of CRC, the location of the primary tumor, molecular markers, age, and radical surgery (15–17). However, there are few studies on the self-reported prognosis of patients.

Therefore, in this study, we conducted a nationwide multi-center cross-sectional study. The involvement of patients with advanced CRC in treatment decision-making and the influencing factors were evaluated. Moreover, the prognosis of advanced CRC patients was comprehensively assessed by using patient self-report efficacy. Additionally, the influencing factors of patient self-reported efficacy were also analyzed. Our findings may provide evidence for further improvement of treatment in patients with advanced CRC.

## 2 Materials and methods

### 2.1 Study design

This is a nationwide multicenter cross-sectional study and the details of the study design have been published (18). In brief, a multi-stage sampling method was used to identify the 19 tertiary hospitals (10 tertiary cancer hospitals and 9 tertiary general hospitals) in China from March 2020 to March 2021. Firstly, two cities were randomly selected from seven administrative regions in East China, North China, Central China, South China, Northeast China, Southwest China, and Northwest China; subsequently, one tertiary cancer hospital or tertiary general hospital in each city was selected as the research center. This study was approved by the Medical Ethics Committee of Henan Cancer Hospital (No. 2019273) and also by the Ethics Committee of all other participating hospitals subsequently. Informed consent was obtained from each participant.

### 2.2 Patients

As previously described (18), it was estimated that more than 4445 advanced CRC patients would be enrolled. The total sample size was proportionally allocated to each region according to its population size and based on the estimated sample size, patients should be recruited from each region. A total of 4,589 inpatients with advanced CRC who had stage III or IV CRC across seven geographic regions of China’s mainland were included in this study from March 2020 to March 2021. Fifty-three cases were excluded due to a lack of essential information for the current analysis. Ultimately, 4,533 cases were included.

The Tumor Lymph Node Metastasis (TNM) staging system by the American Joint Committee on Cancer (AJCC) was used to identify the eligible subjects. The inclusion criteria were as follows: 1) CRC patients with TNM stage III or IV; 2) patients aged more than 18 years; 3) patients with normal cognitive ability; 4) patients willing to participate in the research and signed the informed consent. Exclusion criteria: patients with severe physical, cognitive, and/or verbal limitations were excluded.

### 2.3 Data collection

A self-designed questionnaire was used to collect the demographic and clinical characteristics, patient awareness of CRC risk factors, involvement in treatment decision-making, medical experience, treatment methods, treatment efficacy, etc. Before the formal survey, a preliminary survey was conducted among 50 CRC patients at the Henan Cancer Hospital and the First Affiliated Hospital of Baotou Medical College to evaluate the validity and reliability of the questionnaire. Then we revised the questionnaire based on the preliminary results. The final questionnaire consisted of four parts in 9 pages. To ensure the study quality, all investigators received standard training. Questionnaires were filled out face-to-face by the investigators, with a mean survey time of 20 min.

Involvement in treatment decision-making was classified into “full treatment decision-making by patients, joint treatment decision-making by patients and their family members, treatment decision-making by family members, and treatment decision-making by doctors”. The evaluation of the efficacy was mainly based on the patient’s self-report, and was designed as “poor, good, or stable (unchanged)”. The awareness of CRC risk factors was evaluated by multiple-choice questions, namely, “What do you think are the risk factors for CRC before diagnosis?”, “What do you think is the appropriate CRC screening method before diagnosis?”, and “Which method do you use to acquire knowledge about CRC?”, which contain 10, 6, and 10 options, respectively. Any selected option was scored as 1 point, while “don’t know” or “seldom see” options were scored as 0 points. Therefore, the total score of each question was 9 points, 5 points, and 9 points, respectively.

The clinical characteristics and treatment methods in the questionnaire were provided by doctors according to patient medical records of diagnosis and treatment, mainly including clinical stage, metastasis, and the treatment and surgical methods during the treatment process.

### 2.4 Quality control and data processing

The questionnaire was designed in standard Chinese. To avoid possible biases, the survey was conducted face-to-face by trained local investigators who were fluent in standard Chinese and the local language to ensure an adequate understanding of the questions by the study participants. To ensure the consistency and the quality of the questionnaire distribution process, in addition to standard training, each investigator had an implementation manual for timely review and to ensure that all processes were carried out following the standard steps and procedures specified in the manual. After the completion of the questionnaire by the investigators, members of the project team would review the questionnaire, and if any missing information or obvious logical errors were found, verification with the patients was required. After data collection, double data entry and validation were performed by two investigators using Epidata software V.3.1.

### 2.5 Statistical analysis

All statistical analyses were performed by using SASV.9.4 software. Continuous variables were expressed as mean and standard deviations and categorical variables were expressed as absolute frequencies and percentages. Univariate analysis was performed by using the t-test, analysis of variance, and chi-square test. The variables with P<0.1 in the univariate analysis were included in the multinomial logistic regression analysis. For treatment decision-making, full decision-making by patients served as the reference. For efficacy, the poor treatment effect was used as the reference. Unordered multinomial logistic regression was used to evaluate influencing factors. All statistical analyses were two-sided with a significance level of 0.05.

## 3 Results

### 3.1 Demographic characteristics

A total of 4533 patients with advanced CRC were enrolled. Their demographic characteristics are shown in **Table 1**. The average age at diagnosis of enrolled patients was 58.7 ± 11.81 years old, and there were 2694 males (59.4%) and 1839 females (40.6%). A total of 2063 (45.5%) patients had colon cancer, while 2470 (54.5%) cases were with rectal cancer. About 17.9% of patients were unemployed, 29% had an education level of primary school and below, 98.9% had medical insurance, 57.5% had an average annual family income of less than 50,000 Yuan, and, 56.2% had medical costs not covered by themselves or their spouses.

**Table 1:** Demographic characteristics of patients with advanced colorectal cancer.

Characteristics	Total number of cases, n (%)	Colon cancer, n (%)	Rectal cancer, n (%)	P-value
Gender				0.329
Male	2694 (59.4)	1210 (58.7)	1484 (60.1)	
Female	1839 (40.6)	853 (41.3)	986 (39.9)	
Age at diagnosis, years (mean ± SD)	58.7 ± 11.81	58.3 ± 12.02	59.0 ± 11.62	0.136
<50	922 (20.3)	443 (21.5)	479 (19.4)	
50~64	2134 (47.1)	972 (47.1)	1162 (47.0)	
≥65	1477 (32.6)	648 (31.4)	829 (33.6)	
Occupation				0.007
Government and public sector personnel	1918 (42.3)	925 (44.8)	993 (40.2)	
Service workers, migrant workers, and individuals	1805 (39.8)	789 (38.2)	1016 (41.1)	
Unemployment, layoffs, etc.	810 (17.9)	349 (16.9)	461 (18.7)	
Marital status				0.753
Married	4266 (94.1)	1939 (94.0)	2327 (94.2)	
Other	267 (5.9)	124 (6.0)	143 (5.8)	
Level of education†				<0.001
Elementary school and below	1314 (29.0)	539 (26.2)	775 (31.4)	
Middle or high school	2495 (55.1)	1157 (56.2)	1338 (54.2)	
University and above	721 (15.9)	364 (17.7)	357 (14.5)	
Medical insurance				0.823
No	51 (1.1)	24 (1.2)	27 (1.1)	
Yes	4482 (98.9)	2039 (98.8)	2443 (98.9)	
Average annual income (Yuan)				<0.001
<50000	2607 (57.5)	1091 (52.9)	1516 (61.4)	
5000-99999	1278 (28.2)	654 (31.7)	624 (25.3)	
≥100000	648 (14.3)	318 (15.4)	330 (13.4)	
Region				<0.001
East China	1312 (28.9)	570 (27.6)	742 (30.0)	
North China	556 (12.3)	254 (12.3)	302 (12.2)	
South China	650 (14.3)	340 (16.5)	310 (12.6)	
Central China	675 (14.9)	271 (13.1)	404 (16.4)	
Northeast China	363 (8.0)	196 (9.5)	167 (6.8)	
Southwest China	651 (14.4)	278 (13.5)	373 (15.1)	
Northwest China	326 (7.2)	154 (7.5)	172 (7.0)	
Bearer of the cost of treatment†				0.029
Payment by patients themselves and their spouses	1981 (43.8)	937 (45.6)	1044 (42.4)	
Not paid by patients themselves and their spouses	2539 (56.2)	1118 (54.4)	1421 (57.6)	
The score for awareness of risk factors (Mean ± SD)	0.91 ± 1.50	0.98 ± 1.58	0.85 ± 1.43	0.003
The score for awareness of the screening method (Mean ± SD)	0.26 ± 0.69	0.29 ± 0.74	0.23 ± 0.65	0.007
The score for awareness of the treatment method (Mean ± SD)	1.17 ± 1.58	1.23 ± 1.61	1.12 ± 1.56	0.022

^†^The total number varies due to missing values.

### 3.2 Clinical characteristics

The clinical characteristics of the 4533 enrolled patients are listed in **Table 2**. Among them, 45.9% were recruited from specialized tumor hospitals and 54.1% were from general hospitals. 18.3% of the patients visited more than 3 hospitals to further confirm their disease status, 87.6% found suspected symptoms themselves, while only 5.8% had hospital visits based on abnormal results during regular health examinations. For tumor stage, 76.8% of the patients were classified as stage III or IV CRC, and 37.9% had metastasis at their first diagnosis, of whom 14.0% had liver metastasis. In terms of treatment methods, 32.4% of the patients received surgery combined with chemotherapy, 13.1% underwent surgery combined with chemotherapy and targeted therapy, and 9.8% were treated with surgery alone.

**Table 2:** Clinical characteristics of patients with advanced colorectal cancer.

Characteristics	Total number of cases, n (%)	Colon cancer, n (%)	Rectal cancer, n (%)	P-value
Type of visited hospital				0.094
Specialized cancer hospital	2083 (45.95)	920 (44.6)	1163 (47.1)	
General Hospital	2450 (54.05)	1143 (55.4)	1307 (52.9)	
Number of visited hospitals†				0.119
1	1328 (29.3)	609 (30.0)	719 (29.7)	
2	2290 (50.52)	1017 (50.1)	1273 (52.6)	
≥3	830 (18.31)	403 (19.9)	427 (17.7)	
Reason for the first hospital visit†				<0.001
Observation of suspected symptoms by patients themselves	3969 (87.56)	1722 (84.0)	2247 (91.4)	
Physical examination findings	264 (5.82)	155 (7.6)	109 (4.4)	
Detection of CRC during screening or treatment of other diseases	275 (6.07)	173 (8.4)	102 (4.1)	
Current treatment phase†				0.012
Treatment had not yet been started	155 (3.42)	65 (3.2)	90 (3.6)	
The first treatment was not replaced	2057 (45.38)	925 (44.9)	1132 (45.8)	
First treatment replacement regimen	524 (11.56)	241 (11.7)	283 (11.5)	
The stage of treatment after relapse	1124 (24.8)	554 (26.9)	570 (23.1)	
Periodic review phase	669 (14.76)	275 (13.3)	394 (16.0)	
Clinical staging at initial diagnosis†				<0.001
Stage I/II	871 (19.21)	405 (20.3)	466 (19.7)	
Stage III	1948 (42.97)	753 (37.8)	1195 (50.6)	
Stage IV	1535 (33.86)	834 (41.9)	701 (29.7)	
Metastasis at diagnosis†				<0.001
No metastasis	2817 (62.14)	1149 (56.0)	1668 (67.8)	
With liver metastasis only	635 (14.01)	362 (17.6)	273 (11.1)	
With lung metastasis only	178 (3.93)	70 (3.4)	108 (4.4)	
With both liver and lung metastases	191 (4.21)	98 (4.8)	93 (3.8)	
Metastases in other sites or multiple metastases throughout the body	689 (15.2)	372 (18.1)	317 (12.9)	
Time since cancer diagnosis (months)				0.347
<12	2543 (56.1)	1173 (56.9)	1370 (55.5)	
>=12	1990 (43.9)	890 (43.1)	1100 (44.5)	
Treatment modality				<0.001
Surgery + chemotherapy	1470 (32.43)	769 (37.3)	701 (28.4)	
Surgery + chemotherapy + targeted therapy	595 (13.13)	365 (17.7)	230 (9.3)	
Surgery + chemotherapy + radiotherapy	381 (8.41)	48 (2.3)	333 (13.5)	
Surgery	442 (9.75)	206 (10.0)	236 (9.6)	
Chemotherapy + targeted therapy	211 (4.65)	121 (5.9)	90 (3.6)	
Chemotherapy	164 (3.62)	82 (4.0)	82 (3.3)	
Surgery + radiotherapy + chemotherapy + targeted therapy	171 (3.77)	50 (2.4)	121 (4.9)	
Radiation therapy + chemotherapy	128 (2.82)	6 (0.3)	122 (4.9)	
Others	971 (21.42)	416 (20.2)	555 (22.5)	

^†^The total number varies due to missing values.

### 3.3 Involvement in treatment decision-making

**Figure 1:**
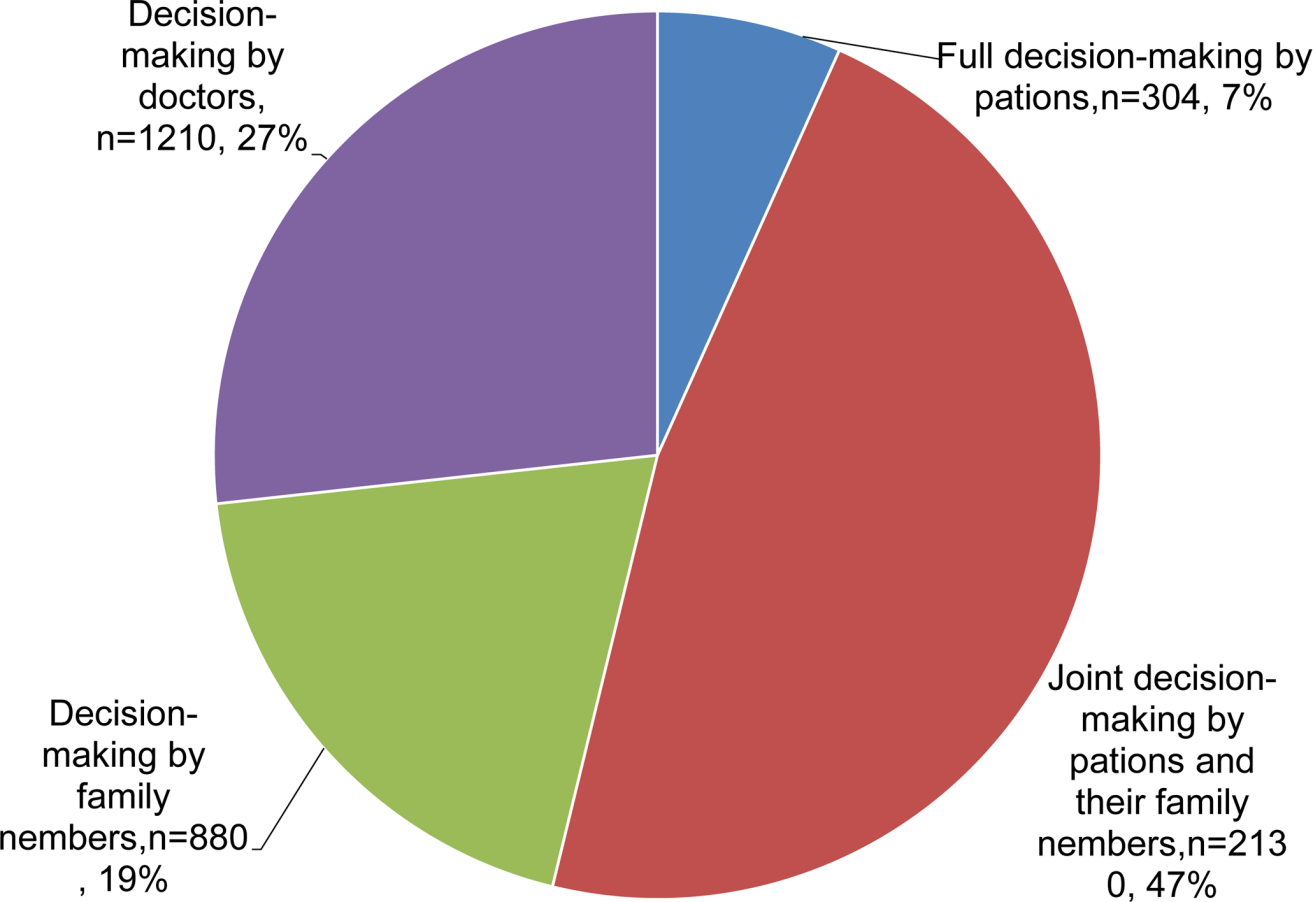
Pie chart showing the involvement in treatment decision-making in patients with advanced colorectal cancer.

In terms of patient involvement in treatment decision-making, 7.0% of patients had full responsibility for treatment decision-making throughout treatment, 47.0% of patients shared treatment decision-making with family members, 19% of the patients relied exclusively on their family members for decision-making, while 27% of the patients left the responsibility of treatment decision-making entirely to their physicians (**Figure 1**).

### 3.4 Univariate and multinomial analysis of patient involvement in treatment decision-making

In univariate analysis, factors associated with involvement in treatment decision-making included gender, age at diagnosis, time since cancer diagnosis, occupation, marital status, education level, average annual family income, treatment cost burden, awareness of CRC-related factors, the type of visited hospital, the number of hospital visits, the clinical stage at diagnosis, and the treatment method (p<0.01 for all) (**Table 3**).

**Table 3:** Univariate analysis of factors affecting patient involvement in treatment decision-making.

Factor	Total number of cases, n(%)	Full decision-making by patients, n(%)	Joint decision-making by patients and their family members, n (%)	Decision-making by family members, n (%)	Decision-making by doctors, n (%)	P
Gender						<0.001
Male	2694 (59.4)	218 (71.7)	1276 (59.9)	463 (52.6)	733 (60.6)	
Female	1839 (40.6)	86 (28.3)	854 (40.1)	417 (47.4)	477 (39.4)	
Age at diagnosis, years						<0.001
<50	922 (20.3)	103 (33.9)	452 (21.2)	87 (9.9)	279 (23.1)	
50~64	2134 (47.1)	141 (46.4)	1063 (49.9)	335 (38.1)	591 (48.8)	
≥65	1477 (32.6)	60 (19.7)	615 (28.9)	458 (52.0)	340 (28.1)	
Time since cancer diagnosis, months						0.001
<12	2543 (56.1)	162 (53.3)	1136 (53.3)	526 (59.8)	713 (58.9)	
≥12	1990 (43.9)	142 (46.7)	994 (46.7)	354 (40.2)	497 (41.1)	
Patient occupation						<0.001
Government and public sector personnel	1918 (42.3)	153 (50.3)	934 (43.8)	347 (39.4)	480 (39.7)	
Service workers, migrant workers, and individuals	1805 (39.8)	118 (38.8)	855 (40.1)	327 (37.2)	502 (41.5)	
Unemployment, layoffs, etc	810 (17.9)	33 (10.9)	341 (16.0)	206 (23.4)	228 (18.8)	
Marital status						0.012
Married	4266 (94.1)	277 (91.1)	2023 (95.0)	816 (92.7)	1142 (94.4)	
Other	267 (5.9)	27 (8.9)	107 (5.0)	64 (7.3)	68 (5.6)	
Level of education†						<0.001
Elementary school and below	1314 (29.0)	51 (16.8)	502 (23.6)	401 (45.6)	358 (29.6)	
Middle or high school	2495 (55.1)	149 (49.0)	1263 (59.3)	409 (46.5)	668 (55.3)	
University and above	721 (15.9)	104 (34.2)	364 (17.1)	70 (8.0)	182 (15.1)	
Average annual household income (Yuan)						<0.001
<50000	2607 (57.5)	140 (46.1)	1134 (53.2)	592 (67.3)	738 (61.0)	
50000~99999	1278 (28.2)	77 (25.3)	667 (31.3)	203 (23.1)	327 (27.0)	
≥100000	648 (14.3)	87 (28.6)	329 (15.4)	85 (9.7)	145 (12.0)	
Bearer of the cost of treatment†						<0.001
Payment by patients themselves and their spouses	1981 (43.8)	174 (57.4)	1005 (47.3)	231 (26.3)	567 (47.1)	
Not paid by patients themselves and their spouses	2539 (56.2)	129 (42.6)	1121 (52.7)	647 (73.7)	637 (52.9)	
The score for awareness of risk factors (Mean ± SD)	0.91 ± 1.50	1.13 ± 1.65	0.99 ± 1.57	0.82 ± 1.43	0.78 ± 1.38	<0.001
The score for awareness of the screening method (Mean ± SD)	0.25 ± 0.70	0.34 ± 0.73	0.30 ± 0.75	0.19 ± 0.60	0.20 ± 0.64	<0.001
The score for awareness of the treatment method (Mean ± SD)	1.17 ± 1.58	1.43 ± 1.55	1.25 ± 1.59	1.13 ± 1.51	1.02 ± 1.62	<0.001
Type of hospital visited						<0.001
Specialized cancer hospital	2083 (46.0)	124 (40.8)	933 (43.8)	269 (30.6)	754 (62.3)	
General hospital	2450 (54.0)	180 (59.2)	1197 (56.2)	611 (69.4)	456 (37.7)	
Number of visited hospitals†						<0.001
1	1328 (29.9)	85 (28.1)	609 (29.3)	345 (40.1)	286 (23.9)	
2	2290 (51.5)	140 (46.4)	1070 (51.4)	391 (45.5)	686 (57.4)	
≥3	830 (18.7)	77 (25.5)	403 (19.4)	124 (14.4)	224 (18.7)	
Clinical staging at initial diagnosis†						0.028
Stage I/II	871 (20.0)	54 (18.6)	430 (21.0)	186 (22.3)	198 (16.9)	
Stage III	1948 (44.7)	126 (43.3)	895 (43.6)	380 (45.6)	543 (46.4)	
Stage IV	1535 (35.3)	111 (38.1)	726 (35.4)	268 (32.1)	428 (36.6)	
Treatment modality						<0.001
Surgical treatment	442 (9.8)	17 (5.6)	186 (8.7)	153 (17.4)	85 (7.0)	
Non-surgical treatment	4072 (89.8)	284 (93.4)	1939 (91.0)	719 (81.7)	1122 (92.7)	
Palliative care	19 (0.4)	3 (1.0)	5 (0.2)	8 (0.9)	3 (0.2)	

^†^The total number varies due to missing values.

Multivariate logistic regression analysis was conducted by using full treatment decision-making by patients served as the reference. The results showed that gender, age, education level, annual family income, marital status, treatment cost burden, type of hospitals visited, and treatment methods were significantly related to involvement in treatment decision-making (**Table 4**). In detail, males were more likely to be solely responsible for treatment decision-making (OR 0.55 to 0.64). Patients under 50 years of age were more dominant in treatment decision-making compared to those over 65 years of age (OR 0.19 to 0.46). Compared with patients with an education level of university or above, patients with elementary school or less education level were less involved in making treatment decisions and were more likely to have family members or doctors make treatment decisions (OR 2.55 to 4.34). Patients with middle or high school education levels were more likely to make treatment decisions with family members (OR=1.96,95% CI=1.398-2.734). Patients with an average annual household income between 50,000 Yuan and 100,000 Yuan were more likely to make treatment decisions jointly with family members or by family members and physicians than patients with an average annual household income greater than 100,000 Yuan (OR 1.53-1.9). Married patients preferred shared treatment decision-making with family members or full treatment decision-making by family members than unmarried, divorced, or widowed patients (OR 1.7-1.88). Patients who paid treatment costs by themselves and their spouses were less likely to let family members make treatment decisions (OR=0.46, 95% CI=0.336-0.692). Patients from specialized cancer hospitals were more likely to share treatment decision-making with family members or follow physicians’ treatment decisions compared to those from general hospitals (OR=1.43-2.98). Patients with surgical treatment were more likely to make treatment decisions jointly with or solely by family members compared to those with palliative care (OR=8.49-9.23).

**Table 4:** Multinomial analysis of factors affecting patient involvement in treatment decision-making.

Factor	Joint decision-making by patients and their family members		Decision-making by family members		Decision-making by doctors	
	OR (95%CI)	*P*	OR (95%CI)	*P*	OR (95%CI)	*P*
Gender						
Male	0.55 (0.409,0.73)	<.0001	0.47 (0.343,0.647)	<.0001	0.64 (0.47,0.863)	0.0036
Female	1	—	1	—	1	—
Age at diagnosis, years						
<50	0.45 (0.298,0.664)	<.0001	0.19 (0.121,0.305)	<.0001	0.46 (0.3,0.695)	0.0003
50~64	0.73 (0.512,1.029)	0.5305	0.41 (0.281,0.589)	0.6177	0.7 (0.488,1.012)	0.7741
≥65	1	—	1	—	1	—
Level of education						
Elementary school and below	1.87 (1.17,2.982)	0.143	4.34 (2.523,7.449)	<.0001	2.55 (1.563,4.174)	0.0042
Middle or high school	1.96 (1.398,2.734)	0.0083	2.65 (1.735,4.051)	0.1213	2.02 (1.406,2.9)	0.1012
University and above	1	—	1	—	1	—
Average annual household income						
<50000	1.13 (0.78,1.643)	0.3456	1.12 (0.717,1.749)	0.5746	1.47 (0.98,2.194)	0.7055
50000~99999	1.71 (1.187,2.475)	0.0017	1.53 (0.979,2.387)	0.0365	1.9 (1.273,2.833)	0.0054
≥100000	1	—	1	—	1	—
Marital status						
Married	1.88 (1.165,3.019)	0.0097	1.7 (1.004,2.884)	0.0483	1.59 (0.962,2.63)	0.0704
Other	1	—	1	—	1	—
Bearer of the cost of treatment						
Payment by patients themselves and their spouses	0.79 (0.603,1.047)	0.102	0.46 (0.336,0.629)	<.0001	0.81 (0.605,1.081)	0.1509
Not paid by patients themselves and their spouses	1	—	1	—	1	—
Type of visited hospital						
Specialized cancer hospital	1.43 (1.075,1.892)	0.0138	1.08 (0.782,1.482)	0.6507	2.98 (2.205,4.013)	<.0001
General Hospital	1	—	1	—	1	—
Treatment modality						
Surgical treatment	9.23 (1.835,46.391)	0.0047	8.49 (1.785,40.339)	0.001	4.91 (0.87,27.705)	0.0855
Non-surgical treatment	5.83 (1.253,27.114)	0.1207	3.38 (0.773,14.741)	0.7183	4.24 (0.814,22.054)	0.1496
Palliative care	1	—	1	—	1	—

### 3.5 Self-reported efficacy

**Figure 2:**
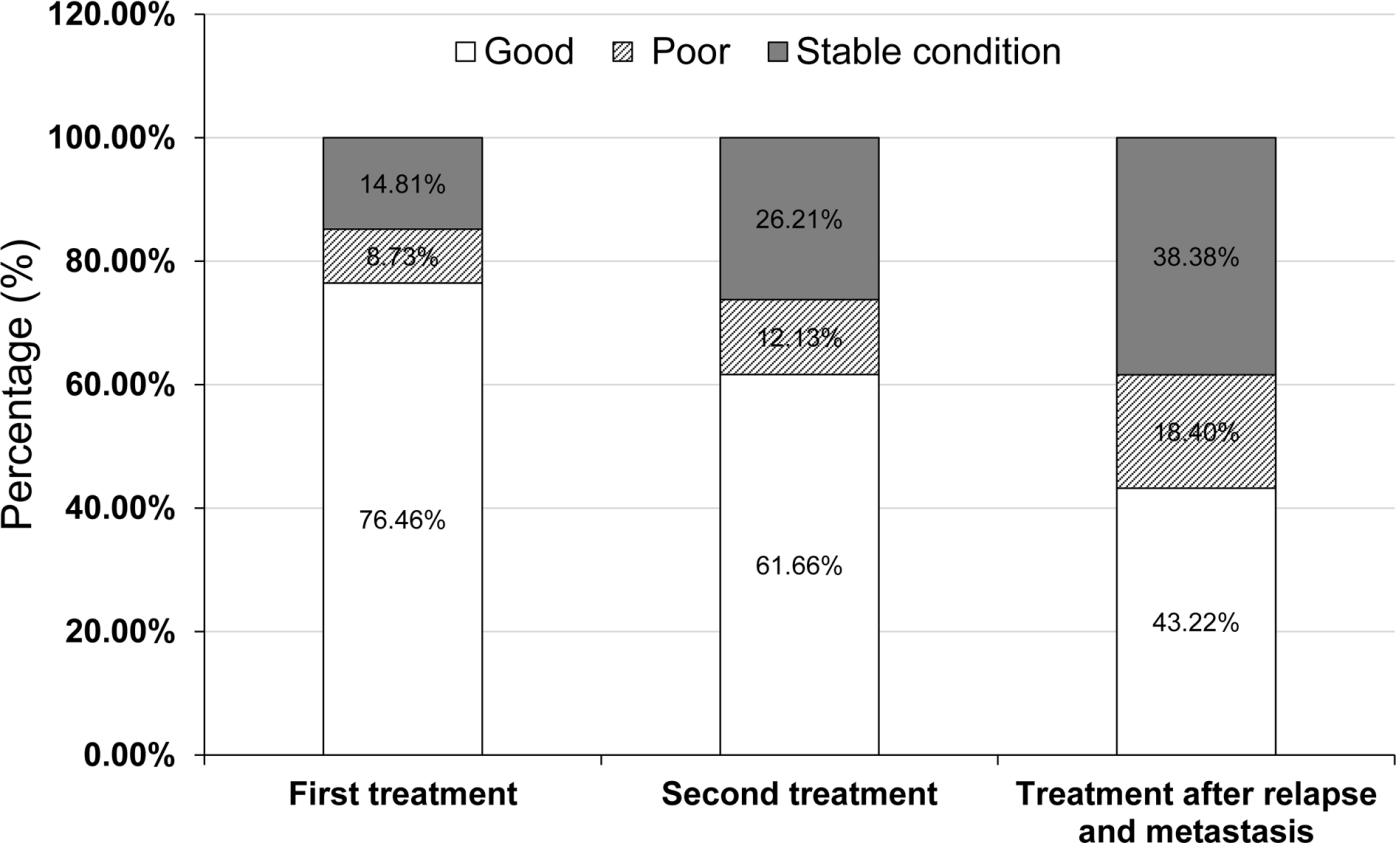
Self-reported efficacy of patients with advanced colorectal cancer.

Self-reported efficacy evaluations were available for 3824 patients. For the first treatment, 76.5% of patients reported efficacy as good, 14.8% reported a stable condition, and 8.7% reported poor efficacy (**Figure 2**). Regarding the efficacy of the second treatment, the percentage of patients with self-reported good efficacy, stable condition, and poor efficacy was 61.7%, 26.2%, and 12.1%, respectively. Regarding the treatment efficacy for patients who had recurrence and metastasis, the percentage of patients with self-reported good efficacy, stable condition, and poor efficacy was 43.2%, 38.4%, and 18.4%, respectively (**Figure 2**).

### 3.6 Univariate and multinomial analysis of factors affecting self-reported efficacy

In univariate analysis, factors associated with self-reported efficacy included age at diagnosis, occupation, education level, average annual family income, region, time since cancer diagnosis, primary site, type of hospital, clinical stage at diagnosis, metastasis status, surgical treatment, chemotherapy, targeted therapy, and involvement in treatment decision-making (**Table 5**).

**Table 5:** Univariate analysis of factors affecting self-reported treatment efficacy.

Factor	Total number of cases, n (%)	Good efficacy,n (%)	Poor efficacy,n (%)	Stable condition,n (%)	P-value
Gender					0.574
Male	2265 (59.2)	1842 (59.2)	270 (58.2)	153 (62.2)	
Female	1559 (40.8)	1272 (40.8)	194 (41.8)	93 (37.8)	
Age at diagnosis					0.001
<50	799 (20.9)	621 (19.9)	116 (25.0)	62 (25.2)	
50~64	1809 (47.3)	1460 (46.9)	223 (48.1)	126 (51.2)	
≥65	1216 (31.8)	1033 (33.2)	125 (26.9)	58 (23.6)	
Patient occupation					0.026
Government and public sector personnel	1617 (42.3)	1337 (42.9)	179 (38.6)	101 (41.1)	
Service workers, migrant workers, and individuals	1527 (39.9)	1214 (39.0)	198 (42.7)	115 (46.7)	
Unemployment, layoffs, etc	680 (17.8)	563 (18.1)	87 (18.8)	30 (12.2)	
Marital status					0.643
Married	3610 (94.4)	2935 (94.3)	440 (94.8)	235 (95.5)	.
Other	214 (5.6)	179 (5.7)	24 (5.2)	11 (4.5)	.
Level of education†					0.014
Elementary school and below	1120 (29.3)	943 (30.3)	113 (24.4)	64 (26.1)	
Middle or high school	2084 (54.5)	1691 (54.3)	259 (55.8)	134 (54.7)	.
University and above	617 (16.1)	478 (15.4)	92 (19.8)	47 (19.2)	.
Average annual household income (Yuan)					0.004
<50000	2204 (57.6)	1821 (58.5)	268 (57.8)	115 (46.7)	
50000~99999	1071 (28.0)	857 (27.5)	134 (28.9)	80 (32.5)	
≥100000	549 (14.4)	436 (14.0)	62 (13.4)	51 (20.7)	
Region					<0.001
Less developed region	1758 (46.0)	1423 (45.7)	248 (53.4)	87 (35.4)	
Developed region	2066 (54.0)	1691 (54.3)	216 (46.6)	159 (64.6)	
Time since cancer diagnosis (months)					<0.001
<12	2092 (54.7)	1857 (59.6)	177 (38.1)	58 (23.6)	
>=12	1732 (45.3)	1257 (40.4)	287 (61.9)	188 (76.4)	
Primary tumor site					0.008
Colon	1767 (46.2)	1410 (45.3)	221 (47.6)	136 (55.3)	
Rectum	2057 (53.8)	1704 (54.7)	243 (52.4)	110 (44.7)	
Type of visited hospital					<0.001
Specialized cancer hospital	1794 (46.9)	1397 (44.9)	259 (55.8)	138 (56.1)	
General Hospital	2030 (53.1)	1717 (55.1)	205 (44.2)	108 (43.9)	
Clinical staging at initial diagnosis†					<0.001
Stage I/II	704 (19.1)	605 (20.1)	73 (16.9)	26 (11.2)	.
Stage III	1683 (45.7)	1431 (47.5)	159 (36.8)	93 (40.1)	.
Stage IV	1292 (35.1)	979 (32.5)	200 (46.3)	113 (48.7)	.
Metastasis at diagnosis†					<0.001
No metastasis	2375 (62.4)	2004 (64.7)	247 (53.3)	124 (50.4)	
With liver metastasis only	534 (14.0)	417 (13.5)	72 (15.6)	45 (18.3)	
With lung metastasis only	146 (3.8)	114 (3.7)	19 (4.1)	13 (5.3)	
With both liver and lung metastases	165 (4.3)	127 (4.1)	25 (5.4)	13 (5.3)	
Metastases in other sites or multiple metastases throughout the body	586 (15.4)	435 (14.0)	100 (21.6)	51 (20.7)	
Surgical treatment†					0.001
No	591 (15.5)	513 (16.5)	53 (11.4)	25 (10.2)	
Yes	3227 (84.5)	2595 (83.5)	411 (88.6)	221 (89.8)	
Chemotherapy†					<0.001
No	491 (12.9)	452 (14.5)	28 (6.0)	11 (4.5)	
Yes	3327 (87.1)	2656 (85.5)	436 (94.0)	235 (95.5)	
Radiotherapy†					0.438
No	2975 (77.9)	2414 (77.7)	372 (80.2)	189 (76.8)	
Yes	843 (22.1)	694 (22.3)	92 (19.8)	57 (23.2)	
Targeted therapy†					<0.001
No	2692 (70.5)	2359 (75.9)	249 (53.7)	84 (34.1)	
Yes	1126 (29.5)	749 (24.1)	215 (46.3)	162 (65.9)	
Involvement in treatment decision-making†					0.018
No	1459 (38.21)	1219 (39.20)	150 (32.47)	90 (36.59)	
Yes	2359 (61.79)	1891 (60.80)	312 (67.53)	156 (63.41)	

^†^The total number varies due to missing values.

^†^Less developed areas: Central, Northwest, Southwest, and Northeast; Developed areas: East, South, Northern.

The multinomial analysis was conducted by using the poor self-reported efficacy as a reference. The results showed that occupation in government and public institutions (OR=1.44, 95%CI=1.044 -1.999), primary education and below (OR=1.57-2.18), annual family income of more than 100,000 Yuan (OR=0.59, 95%CI=0.341-1.005), living in less developed regions (OR=0.51-0.78), time since cancer diagnosis for less than 12 months (OR=1.94, 95%CI=1.547-2.43), admission in general hospitals (OR=0.7, 95%CI=0.558-0.881), clinical stage III (OR=1.41, 95%CI=0.961-2.078), surgery (OR=, 1.72, 95%CI=1.225-2.414), chemotherapy (OR=1.69, 95%CI= 1.101-2.598), targeted therapy (OR=2.03, 95%CI=1.585-2.593), and involvement in treatment decision-making (OR=1.33, 95%CI=1.061-1.665) were significantly associated with good self-reported treatment outcomes (all P<0.05, **Table 6**).

**Table 6:** Multinomial analysis of factors affecting self-reported treatment efficacy (with poor efficacy as a reference).

Factor	Total number of cases, n(%)	Stable condition		Good efficacy	
		OR (95%CI)	*P*	OR (95%CI)	*P*
Patient occupation					
Government and public sector personnel	1617 (42.3)	1.66 (0.949,2.894)	0.2215	1.44 (1.044,1.999)	0.0025
Service workers, migrant workers, and individuals	1527 (39.9)	1.65 (0.983,2.772)	0.185	0.95 (0.708,1.284)	0.0453
Unemployment, layoffs, etc	680 (17.8)	1	—	1	—
Level of education†					
Elementary school and below	1120 (29.3)	2.18 (1.18,4.016)	0.0137	1.57 (1.067,2.323)	0.0360
Middle or high school	2084 (54.5)	1.47 (0.902,2.397)	0.983	1.31 (0.962,1.791)	0.6843
University and above	617 (16.1)	1	—	1	—
Average annual household income (Yuan)					
<50000	2204 (57.6)	0.59 (0.341,1.005)	0.0286	0.83 (0.579,1.198)	0.5391
50000~99999	1071 (28.0)	0.85 (0.508,1.409)	0.5965	0.82 (0.573,1.166)	0.3951
≥100000	549 (14.4)	1	—	1	—
Region					
Less developed region	1758 (46.0)	0.51 (0.36,0.731)	0.0002	0.78 (0.625,0.975)	0.0289
Developed region	2066 (54.0)	1	—	1	—
Time since cancer diagnosis (months)					
<12	2092 (54.7)	0.57 (0.384,0.836)	0.0042	1.94 (1.547,2.43)	<.0001
>=12	1732 (45.3)	1	—	1	—
Type of hospital visited					
Specialized cancer hospital	1794 (46.9)	1.04 (0.729,1.49)	0.8223	0.7 (0.558,0.881)	0.0024
General Hospital	2030 (53.1)	1	—	1	—
Clinical staging at initial diagnosis†					
Stage I/II	704 (19.1)	0.83 (0.402,1.733)	0.235	1.1 (0.695,1.732)	0.6437
Stage III	1683 (45.7)	1.39 (0.774,2.496)	0.0538	1.41 (0.961,2.078)	0.0275
Stage IV	1292 (35.1)	1	—	1	—
Surgical treatment†					
Yes	3227 (84.5)	0.87 (0.492,1.521)	0.6139	1.72 (1.225,2.414)	0.0017
No	591 (15.5)	1	—	1	—
Chemotherapy†					
Yes	3327 (87.1)	1.14 (0.526,2.489)	0.7341	1.69 (1.101,2.598)	0.0164
No	491 (12.9)	1	—	1	—
Targeted therapy†					
Yes	1126 (29.5)	0.49 (0.332,0.717)	0.0003	2.03 (1.585,2.593)	<.0001
No	2692 (70.5)	1	—	1	—
Involvement in treatment decision-making†					
Yes	2359 (61.8)	1.28 (0.900,1.810)	0.1711	1.33 (1.061,1.665)	0.0132
No	1459 (38.2)	1	—	1	—

^†^The total number varies due to missing values.

^†^Less developed areas: Central, Northwest, Southwest, and Northeast; Developed areas: East, South, Northern.

## 4 Discussion

For the first time, we conducted a nationwide multicenter hospital-based survey of patients with advanced CRC. Our results showed that the awareness of CRC-related knowledge such as risk factors, screening methods, and treatment methods was poor in patients with advanced CRC before diagnosis, which was similar to previous studies. For example, Amlani et al. showed that more than half of 2500 people from five European countries who had never received a colonoscopy were unaware that colonoscopy was a screening and prevention tool (14). Mueller et al. showed that only 36.0% of the respondents knew the starting age of CRC screening, and only 8.0% of the respondents answered all the screening knowledge correctly (19).

In this study, only 5.82% of the patients had hospital visits based on abnormal health examination results, while 87.6% found suspected symptoms themselves. Regarding tumor stage, 76.8% of the patients had stage III or IV CRC, and 37.5% had metastasis. These results were consistent with previous studies (20, 21). It has been shown that in countries with long-term and sustainable screening programs for CRC, CRC-related mortality is largely reduced and the diagnostic rate of early-stage CRC is increased (22, 23). Losurdo et al. showed that screening could significantly increase the rate of early diagnosis and surgery in CRC, reduce the incidence of complications, and improve survival outcomes (24). Kanth et al. showed that most of the CRC screening in the United States was based on opportunistic screening, and the goal of the screening rate reached 80.0% in 2018 (25). In China, Urban Cancer Early Diagnosis and Early Treatment Project was carried out in 2012 to screen high-risk groups for CRC in urban areas (26).

In this study, the treatment methods for advanced CRC were mainly surgery, surgery combined with chemotherapy, and, surgery combined with chemotherapy, radiotherapy, and targeted therapy. According to the China guideline for diagnosis and comprehensive treatment of colorectal liver metastases (2020 edition) (27) and Colon Cancer, Version 2.2021, NCCN Clinical Practice Guidelines in Oncology (28), the conventional treatment for CRC is surgery combined with chemotherapy or radiotherapy. Approximately 66.0% and 61.0% of stage II and III colon and rectal patients, respectively, received further treatment with adjuvant chemotherapy and/or radiotherapy (29). For advanced unresectable metastatic CRC, the mainstay of treatment is systemic therapy, such as targeted therapy and immunotherapy (30). In 2021, the American Society of Clinical Oncology (ASCO) and the European Society of Medical Oncology (ESMO) announced many research advances in immune and targeted therapy for advanced CRC (31–34), which greatly improved the survival rate of patients with advanced CRC.

In this study, only 7.0% of patients were solely responsible for treatment decision-making, 47.0% of patients shared treatment decision-making with their family members, and 46.0% of patients had treatment decision-making by solely family members or doctors. These results are consistent with the results of previous studies conducted on Asian patients from Taiwan and the United States (35–37). There are also findings showing that patients are willing to be involved in treatment decision-making, but most patients prefer to make treatment decisions with or by their physicians (38–40). However, it also has been shown that patients in the United States and other developed countries are more willing to make treatment decisions (41, 42). This may be related to the traditional Chinese concept of family and the paternalistic style of doctors. Chinese patients have high trust in doctors, believe that “doctors know the best”, and are willing to have their doctors make treatment decisions (43).

In this study, we found that gender, age at diagnosis, education level, family economic income, marital status, bearer of treatment expenses, type of hospital, and treatment method were independent factors affecting patient involvement in treatment decision-making. Males tended to be more likely to make treatment decisions by themselves, which reflects the dominance of males in the family. Younger patients, those with higher levels of education, and those with higher family income were more independent in making treatment decisions, which was similar to previous studies (44, 45), suggesting that younger and more educated patients are more likely to acquire disease-related information and to be more involved in treatment decision-making. Patients with wealthy families do not have to worry too much about the financial burden of treatment and are willing to have more personal control over their treatment decisions (46). Married patients are more involved in treatment decision-making than those in other marital statuses, which further reflects that Asians have a heavier family concept (37). For patients who paid for treatment costs by themselves and their spouses, family members were less likely to make treatment decisions, mainly because these patients had economic dominance. Compared with general hospitals, doctors from specialized cancer hospitals were more involved in treatment decision-making, which may be related to patients’ higher trust in oncologists. It has been shown that the lack of knowledge of patients and the imbalance of the doctor-patient relationship are the main obstacles for patients to participate in treatment decision-making (47). Clinicians should timely provide information about diseases to patients from the perspective of patients, which has a great impact on patient involvement in treatment decision-making (48, 49). Good and efficient doctor-patient communication is a major factor in improving treatment decision-making satisfaction, treatment compliance, and improved treatment outcomes.

Patient-Reported Outcomes (PROs) refer to any outcomes directly reported by patients, including information related to health, life quality, and functional status (50). PROs may more accurately reflect the physical functioning and emotional well-being of an individual, which cannot be affected by physician interpretation and prejudice (51) and are superior predictors of survival compared with functional status (52). The application of PROs can reduce the symptom burden of CRC patients and improve patients’ quality of life and survival. The evaluation of PRO is mainly through questionnaires (53), including short form-36, European Organization for Research and Treatment of Cancer, the quality of life questionnaire (EORTCQLQC30), and, the European Organization for Research and Treatment of colorectal cancer, the quality of life questionnaire (EORTCQLQ-CR38), etc. (54). In this study, we did not use scales to evaluate PROs. Instead, we used self-reported efficacy, i.e. patients’ subjective feelings after treatment, which was divided into good efficacy, poor efficacy, and stable condition. A total of 3824 patients, mainly those receiving the first treatment, the second treatment, and the treatments following recurrence and metastasis, submitted self-reported efficacy assessments during treatment. With the increase in the number of treatment times, patients reported an increased number of unsatisfactory treatment outcomes, which is also in line with the progression of the disease. Generally, the patient’s quality of life decreases with longer disease duration and advanced disease stages (55).

In this study, occupation, education level, average annual family income, economic level of the region, time since cancer diagnosis, hospital type, clinical stage at diagnosis, surgical treatment, chemotherapy, targeted therapy, and involvement in treatment decision-making were all significant factors affecting self-reported efficacy in patients with advanced CRC. Belachew. et al. found that higher education levels and economic income were associated with higher quality of life (56). McCombie et al. showed that CRC patients over the age of 80 years were always satisfied with the outcome of surgical treatment (57). Moreover, gender, ethnicity, medical insurance, tumor location, stage, metastasis, and other factors are all prognostic factors of CRC (58, 59). In addition, appropriate surgical treatment is essential to control tumor recurrence and metastasis, thus achieving improved survival rates (60). However, in this study, tumor location and metastasis were not independent prognostic factors, which may be related to the fact that all the study participants included in the study were with advanced CRC. In addition, patients who participated in treatment decision-making reported better self-reported treatment outcomes than patients who did not participate in treatment decision-making, possibly because patients who participated in treatment decision-making acquired more knowledge of tumors and had a better quality of life (61).

There are several limitations in this study. First, the role of doctors and nurses in patient involvement in treatment decision-making was not analyzed. Second, this is a cross-sectional study without long-term follow-up. The causal relationship between treatment effects and associated factors cannot be determined. Third, there are some missing values for some variables, which might cause some potential bias. Considering that the highest missing rate of a specific variable was lower than 5%, and the missing rates of most variables were less than 1%, we did not make further adjustments. Last but not least, the treatment effects were self-reported and were only evaluated based on subjective feelings, without using appropriate scales, which may lead to certain biases. In the follow-up work, we will collect more data and conduct a more in-depth analysis of the patient involvement in treatment decision-making and the self-reported efficacy of CRC patients.

In this study, we conducted a nationwide multi-center hospital-based survey of patients with advanced CRC and found that the involvement of patients in treatment decision-making was poor. The vast majority of treatment decisions were made jointly with family members or by family members/physicians. Effective communication between physicians and patients should be further improved. Thus, patients can obtain timely information on CRC and then participate in treatment decision-making. The use of patient self-reported outcomes in clinical practice in China is in its infancy and lacks appropriate measurement tools, which should be further improved in the future.

## Data availability statement

The raw data supporting the conclusions of this article will be made available by the authors, without undue reservation.

## Ethics statement

The study was reviewed and approved by the Medical Ethics Committee of Henan Cancer Hospital (No. 2019273). The patients/participants provided their written informed consent to participate in this study.

## Author contributions

Conception and design: X-FG, H-FX, Y-LQ, LM, and RR. Administrative support: Y-LQ. Provision of study materials or patients: X-FG, H-FX, LL, Y-QY, XZ, X-HW, W-JW, L-BD, S-XD, H-LC, Y-QZ, Y-YL, J-XH, JC, Y-PF, C-YF, X-ML, J-CD, and LM. Collection and assembly of data: Y-QY, XZ, and H-FX. Data analysis and interpretation: X-FG, YL, and H-FX. Manuscript writing: X-FG, H-FX. Final approval of manuscript: All authors. All authors contributed to the article and approved the submitted version.
